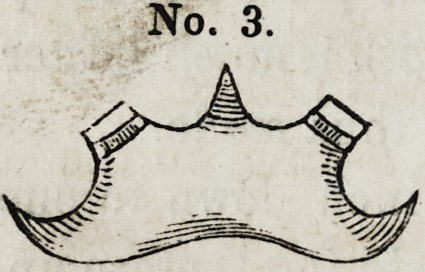# Another European Principle Americanized

**Published:** 1853-10

**Authors:** Wm. M. Hunter

**Affiliations:** Cincinnati, Ohio.


### ARTICLE IV.

*Another European Principle Americanized.* By Wm. M. Hunter, M. D., Cincinnati, Ohio.

In the year eighteen hundred and fifty-one, a gentleman of Cincinnati, during a visit to Europe, employed a Parisian dentist to supply the two inferior bicuspid teeth of the right side, together with the left anterior bicuspid of the same jaw, which operation was performed by carving the teeth and the plate (so to speak) very neatly from the bone of the hippopotamus. The fit was admirable, and the finish unexceptionable; the novelty, however, of the work consisted in the method of retaining it in the mouth, which was as follows: After the piece was fitted, four holes were drilled into the bone on the sides approximating to the natural teeth at a point opposite their necks, into which were inserted cylinders of hard wood, protruding just sufficiently to produce the amount of pressure necessary to retain it in place; unfortunately, however, the natural teeth were separated, and the continual pressure of each new plug rendered necessary by the yielding of the teeth, caused an opening that the artificial teeth would no longer fill, and another contrivance became necessary.

Seeing the principle, and the evil results of its application in this case, it required but very little thought to overcome the difficulty, and the consequence is, an application of the principle to gold plates with entire success, by means of tubes soldered to the plate at proper points and filled with compressed wood.

The advantages in many cases must be apparent to the thinking dentist, but, perhaps, it might not be amiss to enumerate a few.

The fixture is held in place with greater firmness than by means of clasps.

In some instances where I have used clasps, I have also used the tube in combination to give stability for masticating purposes.

The injury to the natural teeth must be much less, owing to the smaller amount of surface in contact.

If decay should take place, it would require but an ordinary filling to restore the tooth.

It prevents that peculiarly disagreeable sensation experienced, particularly in fruit season, upon removing and replacing artificial teeth.

After having tested it for more than a year, I am satisfied that it greatly lessens the chance of decay in those cases where it can be applied, and I have removed the clasps in some old cases with great satisfaction to my patients.

My method of applying the tubes, is as follows: After swaging; the plate, as usual, is tried in the mouth, and an accurate impression of the teeth to be used, is taken over the plate, as recommended by Dr. Arthur, in the American Journal, which will show the exact position of the tooth in its relation to the plate; after which the edge of the plate surrounding the teeth to be made use of, should be doubled or wired, when the tubes may be soldered at their proper points, taking care never to apply pressure to one side of a tooth without some means of counteracting the effect; that means being either a *sufficient number* of natural teeth contiguous to the tooth to be used, a counter tube, an arm of metal, or an artificial tooth, must depend entirely upon the nature of the case.

At times, it is well to tube but one side of the plate and clasp the other; in cases where the crown of the tooth is much larger than the neck, a beautiful application may be thus made.

The tubes should be from one-eighth of an inch down to one line in diameter, and should be filled with whiting before applying heat to prevent them from filling with solder at the time of soldering to the plate. They should be placed upon the plate so carefully, that the mouth of the tube will come in contact with the natural tooth, as it is desirable to have the wood protrude but very slightly beyond the orifice.

Where it can be properly done, the tubes are soldered at the same time the teeth are, as it saves much trouble in fitting; it cannot, however, be very well done where it is designed to fit a tooth over a tube, but can very readily be done where the tube is designod to fill the angle caused by the meeting of the stay and plate, in the incisors and canine teeth, and where a canine is used for a bicuspid, building over the tube with metal to form the inner cusp.

The annexed three cases will serve to exemplify in a measure the application. In case No. 1, the second molar of one side, and the second bicuspid of the other was used, the posterior surface of the first bicuspid (artificial) being used for the counter bearing.

No. 1.

No. 2

In No. 2, the wisdom teeth were used, and after more than a year's wear, I am convinced, that the same service could not have been rendered with clasps, the teeth are conical, notwithstanding which, the *grip* is of surpassing excellence.

No. 3.

No. 3, is a case made for a young lady, with a perfectly symmetrical jaw, and without a separation between the teeth, natural or otherwise. I was not willing to separate for clasps, so concluded, that as *the arch was perfect*, I would try it, and watch the case closely. It was with much reluctance that I applied the tubes in this instance, but time has proven that it may be done in such cases with perfect safety. It is not necessary, that any great amount of force should be exerted in any case to retain it properly in the mouth, an accurate assimilation of the appliance to the natural parts is absolutely required, as the tendency of the plate is decidedly upward.

Hoping that the principle may be of as great benefit to others, as I feel that it has been to my patients, I make it public. Wishing (though I fear not) that it may be used *only* by those dentists who have the skill to properly apply it.

## Figures and Tables

**No. 1. f1:**
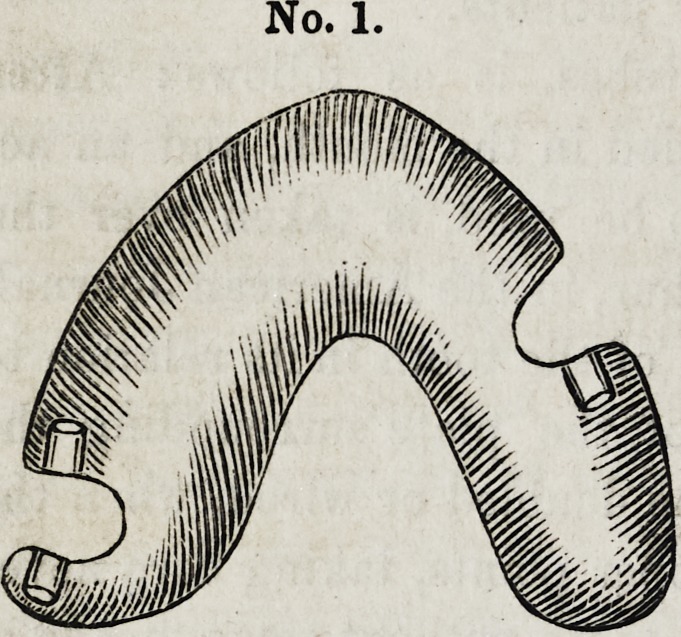


**No. 2 f2:**
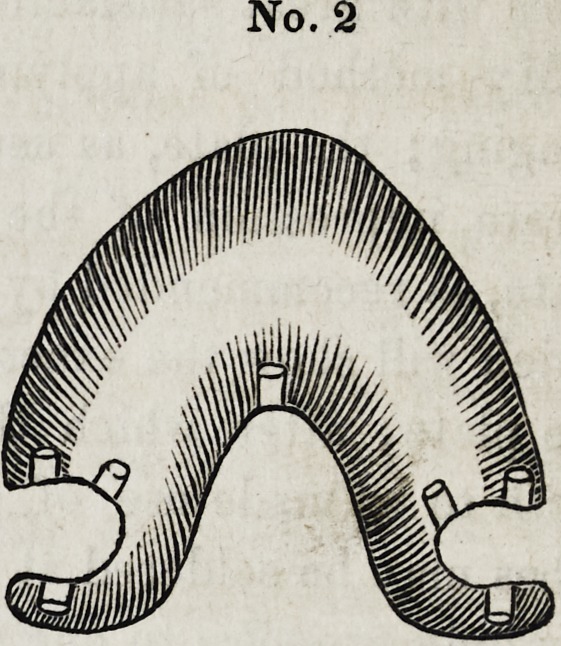


**No. 3. f3:**